# Spontaneous Coronary Artery Dissection in a Male Patient with Takayasu's Arteritis and Antiphospholipid Antibody Syndrome

**DOI:** 10.1155/2013/272963

**Published:** 2013-07-01

**Authors:** Demet Menekşe Gerede, Bağdagül Yüksel, Eralp Tutar, Orhan Küçükşahin, Çağlar Uzun, Kayhan Çetin Atasoy, Nurşen Düzgün, Uğur Bengisun

**Affiliations:** ^1^Department of Cardiology, Ankara University, Faculty of Medicine, Akademik Yerleşke, Sıhhıye, 06100 Ankara, Turkey; ^2^Department of Internal Medicine, Ankara University, Faculty of Medicine, Akademik Yerleşke, Sıhhıye, 06100 Ankara, Turkey; ^3^Department of Internal Medicine-Rheumatology, Ankara University, Faculty of Medicine, Akademik Yerleşke, Sıhhıye, 06100 Ankara, Turkey; ^4^Department of Radiology, Ankara University, Faculty of Medicine, Akademik Yerleşke, Sıhhıye, 06100 Ankara, Turkey; ^5^Department of Vascular Surgery, Ankara University, Faculty of Medicine, Akademik Yerleşke, Sıhhıye, 06100 Ankara, Turkey

## Abstract

We present a case of a 34-year-old male who presented to the emergency ward with fever and abdominal pain. The diagnosis of Takayasu's arteritis and also antiphospholipid syndrome was made during an imaging workup of deep-vein thrombosis. A spontaneous coronary artery dissection was revealed in coronary CT angiography requested for chest pain and dyspnea. The patient was treated medically and discharged on close followup. The concurrence of spontaneous coronary artery dissection with antiphospholipid syndrome and Takayasu's arteritis has not been reported in the previous literature. The possibility of a spontaneous coronary artery dissection should be considered in patients presenting with both diseases.

## 1. Case Report

A 34-year-old Turkish male presented to the emergency ward with a one-week history fever and abdominal pain originated from the epigastric region. History revealed a spiking fever of 38°C accompanied by chills and shivering. The patient was admitted to the Infectious diseases clinic for further investigation of the etiology of fever. Physical examination revealed an oriented and cooperative patient. Blood pressure was 115/80 mmHg, pulse 85/min, respiratory rate 20/min, and body temperature 37,8°C. Cardiopulmonary and abdominal examinations were unremarkable.

Laboratory results were as follows: hemoglobin 11.4 gr/dL, white blood cell count (WBC) 12.7 × 10^9^/L, platelet count 40 × 10^9^/L, erythrocyte sedimentation rate (ESR) 49 mm/h, and C-reactive protein (CRP) 421 mg/L. On peripheral blood smear, thrombocyte count was 50 × 10^9^/L, and there were no atypical cells. Renal and hepatic functions were normal. Microbiological analyses were negative. Since fever increased to 38.5°C on the day after admission, the patient was treated with an empirical antibiotic therapy consisting of piperacillin-tazobactam. 

The patient reported a medical history of deep-vein thrombosis (DVT) and pulmonary thromboembolism three years ago, which had been diagnosed by pulmonary CT angiography and lower-extremity indirect CT venography. There was no information regarding further evaluation of thrombophilia during that period. The patient had been treated with warfarin 5 mg per day for 6 months, which led to resolution of the deep-vein thrombus confirmed by the Doppler ultrasound. 

Further investigation of the etiology of secondary thrombophilia showed strong positivity for lupus anticoagulant at 2.93 (normal <1.2), positive anticardiolipin IgG of  28.9 GPL (normal 1–10 GPL), and a positive anti-beta2-glycoprotein 1 (B2GP1) IgG of 9.3 U/mL (normal 0–5 U/mL). Antinuclear antibody and anti-ds-DNA antibody were negative. The diagnosis of systemic lupus erythematosus (SLE) was ruled out. The patient was treated with acetylsalicylic acid (300 mg/d), hydroxychloroquine (200 mg/d), and enoxaparin (2 × 0.8 cc, SC) with a possible diagnosis of primary antiphospholipid syndrome. 

**Figure 1:**
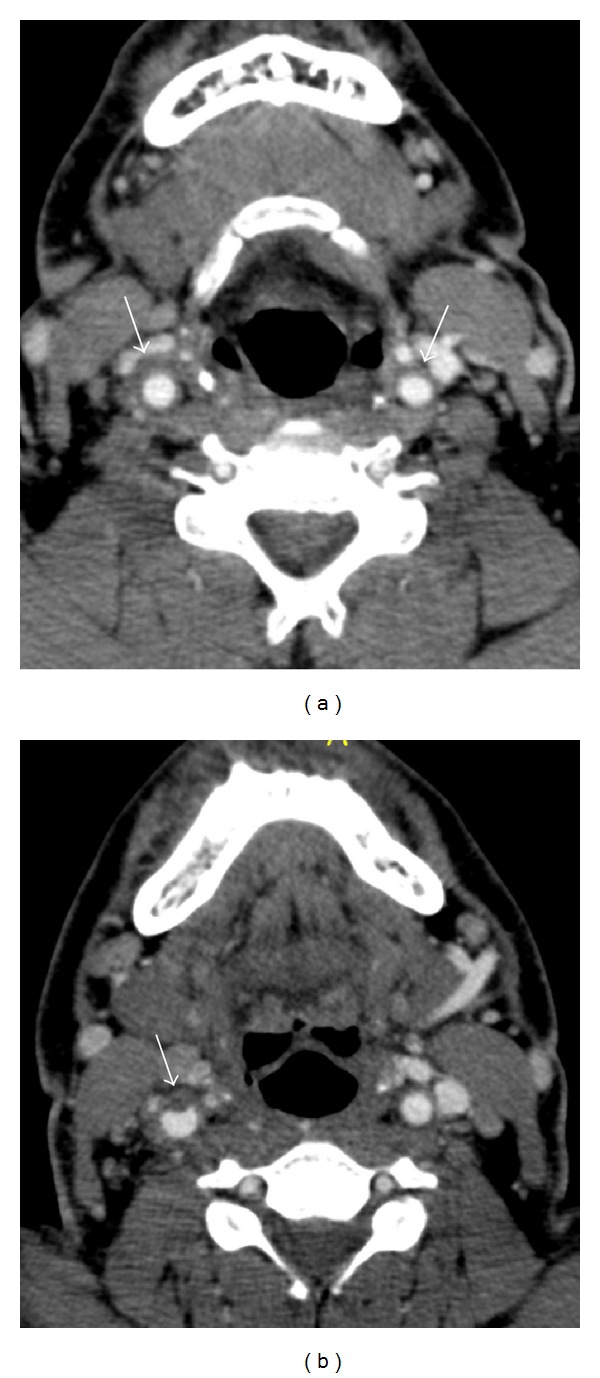
Axial CT angiography sections show wall thickening in both common carotid arteries (arrows, (a)) and a small thrombus protruding into the lumen of the right common carotid artery (arrow, (b)).

**Figure 2:**
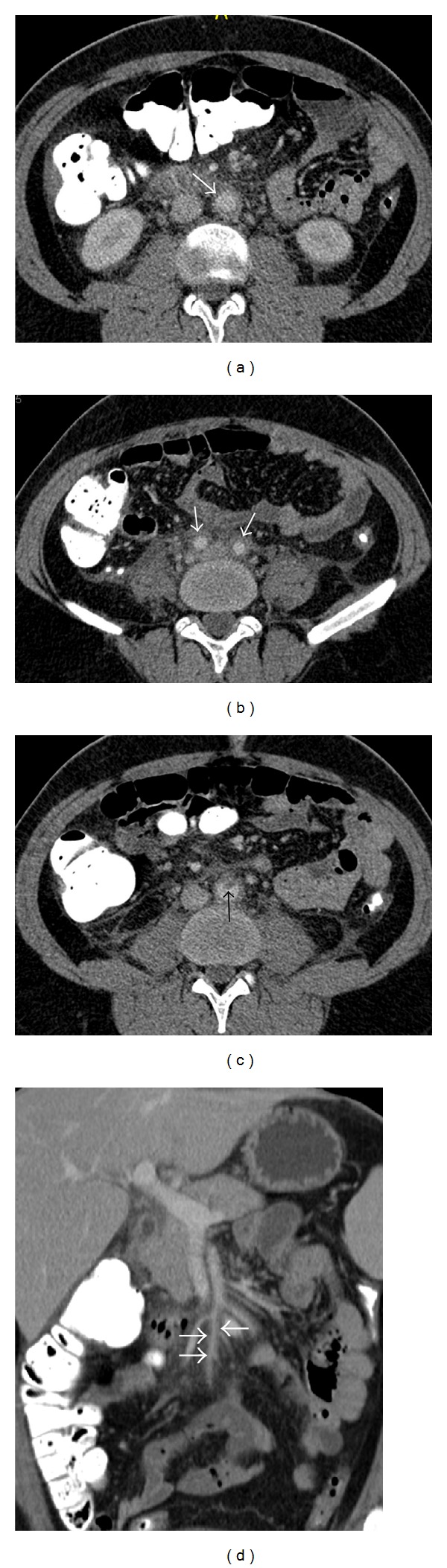
Axial CT angiographic images (a)–(c) show circumferential wall thickening in the abdominal aorta (arrow, (a)) and bilateral iliac arteries (arrows, (b)), as well as a small protruding atheroma in the abdominal aorta (arrow, (c)). Coronal image (d) demonstrates thickening of the wall of the superior mesenteric artery (arrows) and soft-tissue infiltration in the surrounding mesentery.

CT imaging demonstrated bilateral diffuse wall thickening of the common carotid arteries (CCAs) (Figure 1(a)), internal carotid arteries (ICAs), and external carotid arteries (ECAs), a peripheral chronic thrombotic material that measured 3.4 mm in thickness in a short segment just below the bifurcation of the right CCA (Figure 1(b)), diffuse wall thickening of the infrarenal abdominal aorta (Figure 2(a)), iliac arteries (Figure 2(b)), a peripheral thrombus measuring 5 mm in thickness in the aorta just above the aortic bifurcation (Figure 2(c)), wall thickening in the superior (Figure 2(d)) and inferior mesenteric arteries and bilateral renal arteries, and mild soft-tissue thickening in the small-bowel mesentery (Figure 2(d)). 

The patient was subsequently put on 1 mg/kg/day methylprednisolone with the diagnosis of Takayasu's arteritis. No difference greater than 10 mmHg between blood pressures measured on both arms and limbs was noted. 

**Figure 3:**
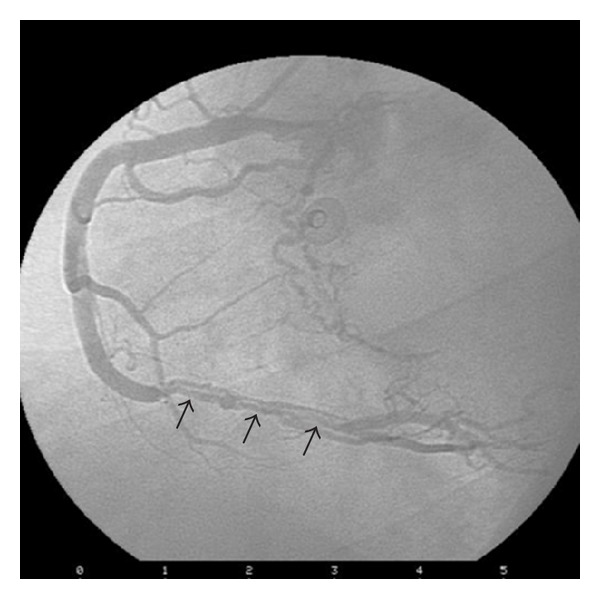
Coronary angiogram shows along coronary dissection at the distal RCA (arrows).

On the third day of corticosteroid therapy, the patient suffered from chest pain and dyspnea. Electrocardiography appeared normal; however, troponin I and CK-MB levels were elevated. He was treated as non-ST segment-elevation myocardial infarction (NSTEMI) with clopidogrel, metoprolol, atorvastatin, and ramipril in addition to his previous therapeutic line of ASA and enoxaparin. Echocardiography demonstrated inferior basal-mid wall hypokinesia. Two days later, when troponin level was further elevated, the patient was referred for an inpatient coronary angiogram, which surprisingly demonstrated a spontaneous dissection extending from the acute marginal branch of the right coronary artery (RCA) to the ostium of the posterior descending artery (PDA) (distal flow TIMI III), with normal left anterior descending (LAD) and circumflex arteries (CX) (Figure 3). 

Percutaneous coronary intervention was not considered because the dissection did not obstruct the blood flow. During the followup at the intensive care unit, chest pain subsided and troponin level fell gradually. A dosage of 15 mg/week methotrexate and folic acid was added to the patient's treatment regimen.

**Figure 4:**
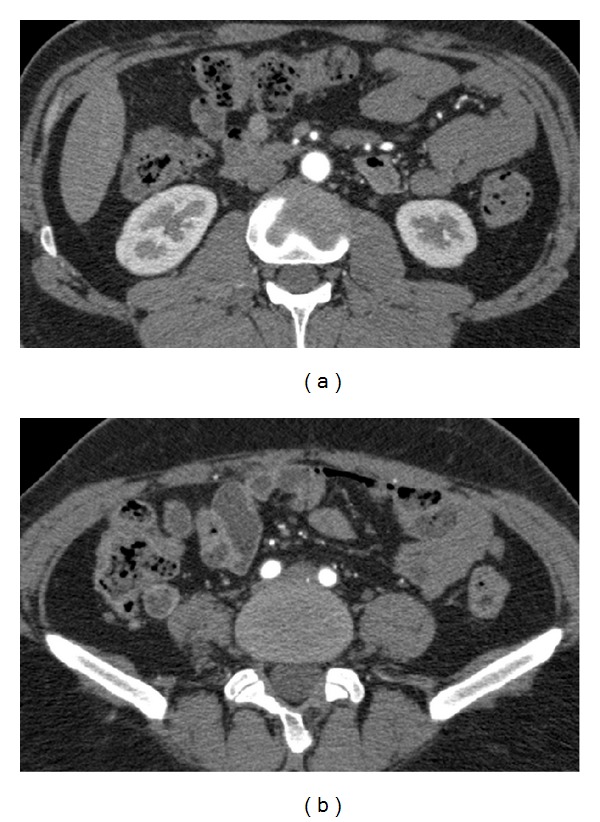
Axial CT angiographic sections show almost normal thickness of the wall of the aorta (a) and both common iliac arteries (b).

On the fourteenth day under corticosteroid therapy, a repeat CT angiography of the aorta demonstrated a decrease in vessel wall thickness and resolution of mesenteric fatty infiltration (Figures 4(a) and 4(b)). 

**Figure 5:**
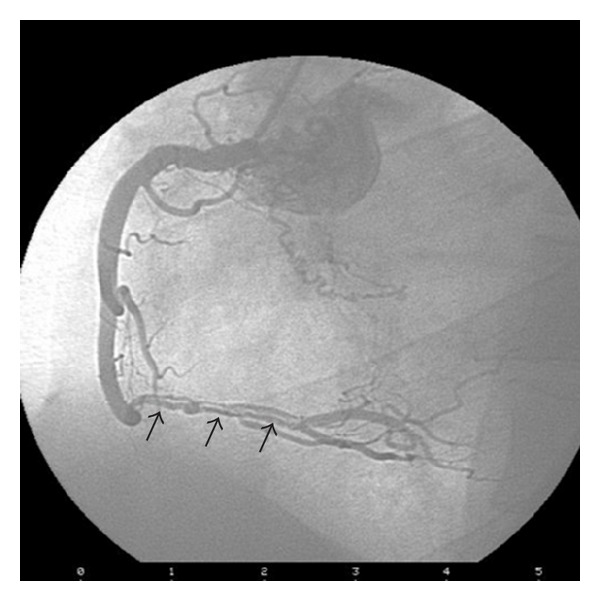
Control coronary angiography performed 15 days later shows no change in the appearance of the dissection (arrows).

A control coronary angiogram performed fifteen days after the first one demonstrated persistence of the dissection found at the distal RCA (Figure 5). 

Laboratory findings showed that ESR was 4 mm/h, CRP was below 0.8 mg/dL, and the thrombocyte count was 280 × 10^9^/L on a 1-week control checkup. The patient was discharged on oral methylprednisolone and methotrexate and advised for follow-up visit for coronary artery dissection. The patient was considered to be in remission for Takayasu's arteritis and planned for a life-long anticoagulation.

## 2. Discussion

Spontaneous coronary artery dissection (SCAD) is an uncommon but clinically relevant cause of acute myocardial infarction in young otherwise healthy patients. The mean age at presentation is 35 to 40 years with a striking predilection for females within this age group. The precise etiology of SCAD remains uncertain. However, reported cases in the literature have focused mainly on female patients in the peripartum or postpartum period, suggesting a potential role of hormonal changes and hemodynamic stress. There have also been cases that have reported connective tissue diseases as potential risk factors for SCAD. Lepper et al. reported a 43-year-old woman who was diagnosed with Churg-Strauss syndrome and died of dissection of all three coronary arteries [1]. An unusual presentation of SCAD has also been reported, which preceded an overt clinical presentation of SLE with positive anticardiolipin IgG antibody [2]. Sharma et al. reported a fatal left coronary artery dissection in a 48-year-old patient diagnosed with SLE [3]. An autopsy study showed extensive aortic dissection involving the arch, along with the thoracic and abdominal aorta in one of ten patients with Takayasu's arteritis [4].

The antiphospholipid syndrome is characterized by thrombotic and obstetric complications in the presence of antiphospholipid antibodies. It occurs in an isolated form or in association with systemic connective tissue diseases, mainly SLE. According to the revised 2006 Clinical Guidelines, a diagnosis of antiphospholipid syndrome requires that the patient has at least one clinical and one laboratory criterion [5]. Clearly, our patient meets more than these criteria by having a history of arterial and venous thrombosis, and laboratory evidence of a strongly positive lupus anticoagulant, positive anticardiolipin IgG, and anti-B2GP1 IgG. The occurrence of SCAD in the presence of antiphospholipid syndrome is rare, and data in the literature are scant. Reed et al. reported a case of spontaneous dissection of the LAD in a 33-year-old female who was diagnosed with antiphospholipid syndrome after a history of recurring abortions [6].

This is the first case in the literature that has reported the occurrence of a coronary artery dissection in the presence of both Takayasu arteritis and antiphospholipid syndrome. This case highlights the importance of considering the possibility of spontaneous coronary artery dissection in patients who present with multiple inflammatory vascular diseases.
